# Serum miRNA modulations indicate changes in retinal morphology

**DOI:** 10.3389/fnmol.2023.1130249

**Published:** 2023-03-03

**Authors:** Riemke Aggio-Bruce, Ulrike Schumann, Adrian V. Cioanca, Fred K. Chen, Samuel McLenachan, Rachael C. Heath Jeffery, Shannon Das, Riccardo Natoli

**Affiliations:** ^1^The John Curtin School of Medical Research, The Australian National University, Acton, ACT, Australia; ^2^The School of Medicine and Psychology, Acton, ACT, Australia; ^3^The Save Sight Institute, Faculty of Medicine and Health, The University of Sydney, Sydney, NSW, Australia; ^4^Centre of Ophthalmology and Visual Science, The University of Western Australia, Perth, WA, Australia; ^5^Lions Eye Institute, Perth, WA, Australia; ^6^Ophthalmology, Department of Surgery, University of Melbourne, East Melbourne, VIC, Australia; ^7^Centre for Eye Research Australia, Royal Victorian Eye and Ear Hospital, East Melbourne, VIC, Australia

**Keywords:** age-related macular degeneration, microRNA, diagnostics, serum miRNAs, neurodegeneration

## Abstract

**Background:**

Age-related macular degeneration (AMD) is the leading cause of vision loss in the developed world and the detection of its onset and progression are based on retinal morphological assessments. MicroRNA (miRNA) have been explored extensively as biomarkers for a range of neurological diseases including AMD, however differences in experimental design and the complexity of human biology have resulted in little overlap between studies. Using preclinical animal models and clinical samples, this study employs a novel approach to determine a serum signature of AMD progression.

**Methods:**

Serum miRNAs were extracted from mice exposed to photo-oxidative damage (PD; 0, 1, 3 and 5 days), and clinical samples from patients diagnosed with reticular pseudodrusen or atrophic AMD. The expression of ~800 miRNAs was measured using OpenArray™, and differential abundance from controls was determined using the HTqPCR R package followed by pathway analysis with DAVID. MiRNA expression changes were compared against quantifiable retinal histological indicators. Finally, the overlap of miRNA changes observed in the mouse model and human patient samples was investigated.

**Results:**

Differential miRNA abundance was identified at all PD time-points and in clinical samples. Importantly, these were associated with inflammatory pathways and histological changes in the retina. Further, we were able to align findings in the mouse serum to those of clinical patients.

**Conclusion:**

In conclusion, serum miRNAs are a valid tool as diagnostics for the early detection of retinal degeneration, as they reflect key changes in retinal health. The combination of pre-clinical animal models and human patient samples led to the identification of a preliminary serum miRNA signature for AMD. This study is an important platform for the future development of a diagnostic serum miRNA panel for the early detection of retinal degeneration.

## 1. Introduction

Age-related macular degeneration (AMD) is the leading cause of irreversible blindness in the elderly population in the western world (Klein et al., 2007; Heath Jeffery et al., 2021). In many cases, the disease is slow progressing and chronic with early stages presenting as relatively asymptomatic (Deloitte Access Economics et al., 2011). Current diagnosis and disease grading is predominantly based on the presentation of small, subretinal lipid deposits called drusen, pigment changes in the central macular region, and self-reported scotomas or ‘blind spots’ (Fine et al., 2000; Klein et al., 2007; Ardeljan and Chan, 2013). In addition, a newly classified form of drusen, reticular pseudodrusen (RPD), has demonstrated clinical significance as a risk factor of progression to one of two forms of late-stage AMD, atrophic AMD (geographic atrophy (GA)) or neovascular AMD (Boddu et al., 2014; Domalpally et al., 2019). However, retinal imaging is used for clinical stratification and therefore is of little use in understanding molecular complexity of AMD, especially when considering that molecular events likely precede histopathological changes. Thus, we propose a combinatorial approach that utilizes both imaging data and serum biomarkers, which is easily explored in pre-clinical animal models as these limit the variability derived from confounding factors such as co-morbidities. The photo-oxidative damage (PD) mouse model, which exposes the rodent retina to bright light (Natoli et al., 2016), has been used extensively to explore molecular facets common to AMD, including oxidative stress, chemokine response, activation of the complement cascade, and immune cell recruitment (Noell et al., 1966; Chen et al., 2004; White et al., 2007; O’Koren et al., 2016; Song et al., 2017). Thus, the model provides a controlled system, enabling detailed investigation into the nuanced molecular changes and their correlations to retinal disease progression especially regarding retinal inflammatory diseases, such as AMD.

MicroRNAs (miRNA) have been shown to be regulated in response to retinal degenerations (Saxena et al., 2015; Chu-Tan et al., 2018; Fernando et al., 2020; Aggio-Bruce et al., 2021; Chu-Tan et al., 2021). MiRNAs are short non-coding RNAs that repress messenger RNA (mRNA) translation by binding to their 3′ untranslated region (Andreeva and Cooper, 2014). As only a short region of complementarity is required for binding, a single miRNA can regulate a large number of targets, often within the same biological pathway (Bandyra Katarzyna et al., 2012). Consequently, miRNAs control almost all biological processes in health and disease and are highly conserved across species (Lu et al., 2008; McCreight et al., 2017). Because of their regulatory role, the modulation of miRNAs also occurs in disease (Askou et al., 2017; Berber et al., 2017; Romano et al., 2017; Chu-Tan et al., 2018), making them ideal targets to determine disease states. Therefore, miRNAs have been explored as a diagnostic tool in many biological systems (Chen et al., 2008; Gilad et al., 2008; Mitchell et al., 2008; Roy and Sen, 2011; Weiland et al., 2012), including the retina (Ertekin et al., 2014; Grassmann et al., 2014; Szemraj et al., 2015; Ménard et al., 2016; Berber et al., 2017; Ren et al., 2017; Romano et al., 2017; Szemraj et al., 2017; ElShelmani et al., 2020). Additionally, miRNAs are abundant in biofluids including tears, saliva, urine, plasma, and serum, making them easily accessible using minimally invasive methods (Chen et al., 2008; Mitchell et al., 2008; McDonald et al., 2011). Further, serum miRNAs are highly stable due to resistance to ribonuclease digestion, integration with RNA-binding proteins and incorporation into extracellular nanovesicles, including exosomes (Chen et al., 2008; Jung et al., 2010; Arroyo et al., 2011; Sanz-Rubio et al., 2018). This high stability and their role in pathology has accelerated research into miRNAs as prognostic tools for disease such as cancer, cardiovascular disease, and neurodegenerative diseases, including retinal degenerations (Chen et al., 2008; Gilad et al., 2008; Lu et al., 2008; Mitchell et al., 2008; McManus and Ambros, 2011; Lukiw et al., 2012; Grasso et al., 2014).

During the last decade, studies into the use of miRNAs as a biomarker for AMD has identified numerous candidates for disease prediction (Grassmann et al., 2014; Szemraj et al., 2015; Ménard et al., 2016; Berber et al., 2017; Ren et al., 2017; Romano et al., 2017). However, due to differing methodologies in the fractionation and collection of samples [serum, tears and plasma (Berber et al., 2017; Roser et al., 2018; Felekkis and Papaneophytou, 2020)], complexities in histological presentation, and the presence of co-morbidities, there is little consensus between studies (Keller et al., 2017). Additionally, a limitation of these studies has been their primary focus on intermediate to late stages of retinal degeneration (Grassmann et al., 2014; Ren et al., 2017; Romano et al., 2017; Elbay et al., 2019; ElShelmani et al., 2020), with little reported for early disease manifestation. However, the development of a diagnostic panel that can identify changes in circulation that reflect early retinal degenerations is essential for successful therapeutic intervention.

In the current study, global serum miRNA analysis was performed to elucidate potential circulating biomarkers for AMD disease progression. Using the pre-clinical PD model, we identified circulating miRNA changes that correlated to changes in overall retinal miRNA expression and abundance. MiRNAs dysregulated as a consequence of progressive degeneration were further associated with inflammatory and apoptosis pathways. Moreover, samples of patients with RPD or GA demonstrated changes in miRNA abundance which correlated to retinal volume. We found the expression of several serum miRNAs, including miR-26a/b-5p, let-7d-5p, miR-19a-3p and miR-574-3p, to be similarly altered in both clinical and PD samples, demonstrating the suitability of this study in overcoming the limitations of current biomarker development. Taken together, these results demonstrate a serum miRNA signature, important for the early detection of retinal degeneration.

## 2. Materials and methods

### 2.1. Animal handling and photo-oxidative damage

All experiments were conducted in accordance with the ARVO Statement for Use of Animals in Ophthalmic and Vision Research. The study was approved by the Australian National University (ANU) Animal Experimentation Ethics Committee (Application ID: 2017/41).

C57BL/6 J mice (Jackson Laboratories, MA, United States) were raised in dim (5 lux), 12-h cyclic light conditions, and were used as dim-reared (DR) controls for this study. To induce PD, C57BL/6 J mice at postnatal day 60 were exposed to 100 K lux natural emission LED light for 1, 3 and 5 days with free access to food and water. Animals were euthanised by administration of CO_2_, and eyes and retinas collected and processed for histological and molecular analysis as described previously (Natoli et al., 2016). Whole blood was collected from the left eye into 1.5 ml Eppendorf tubes (Eppendorf, Germany) by a qualified technician, *via* retro-orbital bleeds using non-heparinised capillary tubes. Serum was separated by first clotting whole blood samples at room temperature for 20 min followed by centrifugation at 1000 x g for 10 min at 4°C.

To control for possible circadian rhythm and gender effects, blood was collected at the same time each day and female animals only were used across the experimental groups. Four mice were used for each sample, with four to six samples per experimental group. Prior to starting this study, we obtained advice from the ANU Statistical Consulting Unit. We further considered the ANU’s ethical requirements of replace, reduce, refine as well as Mead’s resource equation of diminishing returns. Together, these suggested that our animal numbers are sufficient for these experiments. Histology was assessed for each rodent retina and the results averaged within each sample.

### 2.2. Clinical samples, ethics approval and consent to participate

This work adhered to the tenets of the Declaration of Helsinki and was approved by the University of Western Australia Human Research Ethics Committee (protocol: RA/4/1/7916 and 2021/ET000151). Patients were recruited through the Lions Eye Institute (Perth, Western Australia, Australia). Before commencing the study, written informed consent was obtained from the study participants.

Ten patients clinically diagnosed with the presence of reticular pseudodrusen in one or both eyes (early AMD; age: 76 ± 6 years), and five patients with late-stage AMD (geographic atrophy (GA); age: 74 ± 5 years) were included in this study (Supplementary Table S1). RPD diagnosis was made independently by two clinicians (FKC and RCHJ) and was based on the presence of five or more hyporeflective lesions using near-infrared reflectance imaging, which coincided with a hyperreflective deposit above the retinal pigment epithelium (RPE) using spectral domain-optic coherence tomography (SD-OCT) (Wu et al., 2016) imaging. GA was defined by a well demarcated area of outer retinal layer and RPE loss associated with hypoautofluorescence (Holz et al., 2017). The control group consisted of 10 age-matched (72 ± 2 years) participants with no signs of AMD or other retinal pathologies graded by the same clinicians. Venous blood was collected in sterile, dry vacutainer tubes and serum was separated by centrifugation at 2,000 x g for 15 min at 4°C. Serum was stored at-80°C until further processing.

Retinal images were processed using the Heidelberg Spectralis OCT software (Heidelberg Engineering, Germany). The outer nuclear layer (ONL) thickness was measured for each segment at a 1, 2.22, 3.45 mm diameter circles centered around the macular region. The 1 mm volume was used as an indicator of foveal integrity. As this small central zone is often spared in early stages of degeneration, the 3.45 mm volume was also measured as it encompassed the primary lesion on all patients.

### 2.3. TUNEL

Retinal cryosections, cut in the parasagittal plane, were prepared and cell death analyzed using a terminal deoxynucleotidyl transferase dUTP nick end labeling (TUNEL) kit (Roche Applied Science, Germany), as described previously (Natoli et al., 2010). For quantification of photoreceptor cell death, TUNEL^+^ cells in the outer nuclear layer (ONL) were counted along the full length of the retinal section. For each animal, cells were counted in technical duplicate, averaging the counts for each sample group. Significance between the DR control and each PD time point was assessed by one-way ANOVA with Tukey’s multiple comparison post-test.

### 2.4. IBA-1 Immunostaining

Immunohistochemical analysis of retinal cryosections was performed as described previously (Rutar et al., 2015). Immune cells were immunolabeled with primary mouse anti-rabbit IBA-1 (1:1000, Wako, Osaka, Japan) and secondary goat anti-rabbit Alexa 488 (1:500, Thermo Fisher Scientific, MA, United States). Quantification of IBA-1^+^ cells was performed across the superior and inferior retina. The number of IBA-1^+^ cells in the outer retina was quantified as the total counts in the ONL and subretinal space. Significance between the DR control and each PD time point was analyzed by one-way ANOVA with Tukey’s multiple comparison post-test.

Nuclear layers were visualized by staining cryosections with bisbenzimide solution (1:10,000 of a 10 mg/ml stock; Calbiochem, United States). The ONL thickness was measured at increments of 600 μm across the entire retina, including the optic nerve head.

### 2.5. Confocal imaging

Fluorescence was visualized and captured using a ZEISS LSM800 with Airyscan Super-resolution confocal microscope (Carl Zeiss Microscopy, Germany). Images were obtained using uniform gain settings with excitation wavelengths of 488 nm (green; IBA-1 staining), 561 nm (red; TUNEL staining) and 358 nm (bisbenzimide), and processed using ZEISS Zen (blue edition) software.

### 2.6. miRNA extraction and cDNA preparation

Extraction and purification of miRNAs was performed using the miRVana miRNA isolation kit (Thermo Fisher Scientific) following the manufacturer’s instructions. An Agilent 2,100 Bioanalyser with an Agilent small RNA kit (Agilent Technologies, United States) was used to test miRNA purity and concentration.

The OpenArray™ reverse transcription reaction and pre-amplification reaction was performed using the ‘Optimized protocol with low sample input’ (Thermo Fisher Scientific). Reverse transcription and pre-amplification reaction were cycled with a standard PreAmp thermal cycling protocol with 16 cycles on a Veriti 96-well thermal cycler (Applied Biosystems, CA, United States).

### 2.7. OpenArray™

The PreAmp product was diluted 1:20 in 0.1X Tris-EDTA buffer (pH 8.0). OpenArray™ 384-well loading plates were prepared according to the manufacturer’s instructions (Thermo Fisher Scientific). TaqMan™ OpenArray™ Rodent/Human MicroRNA panels were loaded using the OpenArray™ AccuFill™ loader system. Three samples were run on each Array, running four Arrays simultaneously. Post-run, quality control (QC) images were assessed for sample and plate inconsistencies and run quality. Arrays that did not pass QC were repeated.

### 2.8. miRNA OpenArray™ data processing

Both human and mouse miRNA OpenArray™ data were processed as follows: Ct data was filtered by first assigning an “undetermined” label to OpenArray™ wells that had amplification score < 1.24, Ct value >30 or were found outside the top/bottom 10% quantile of each group. MiRNAs with “undetermined” expression in more than 20% of the samples were subsequently removed (Supplementary Figure S1A). From a total of 747 miRNA assayed on the mouse array cards and 715 assayed on the human cards, 199 and 248 were retained after filtering, respectively. Remaining Ct values were imported into the HTqPCR R (Dvinge and Bertone, 2009) packages and normalized using rank invariant miRNAs as reference genes (Mar et al., 2009) then transferred to the *limma* (Ritchie et al., 2015) package for statistical modeling (Supplementary Figure S1B). Linear models were built using the lmFit function, then moderated t-statistics were computed using empirical Bayes moderation (eBayes function) and *p*-values and fold changes were extracted with top Table. The need for inclusion of covariates (experimental batch (array), age, sex) into linear regression models was assessed by (Klein et al., 2007) visually examining the association of each covariate with sample clusters identified by principal component analysis (Supplementary Figure S1C; Heath Jeffery et al., 2021) by observing the effect each covariate has on the distribution of p-values (Supplementary Figure S1D). Both the mouse and human data sets required adjustments for experimental batch, and additional corrections for age were necessary for the latter (Supplementary Figure S1C). Chord diagrams were generated using the circlize R package (Gu et al., 2014) and all other plots were generated using ggplot2 in R unless otherwise indicated.

### 2.9. Network and pathway analysis

The targets of differentially accumulated miRNAs in the 5 day PD samples and the GA patient samples were determined using miRNet (Chang et al., 2020) v2.0 using miRTarBase v8.0 genes as targets. The target list was filtered for interactions validated by HITS/PAR-CLIP and/or reporter assays. The DAVID functional annotation tool (Huang et al., 2009; Sherman et al., 2022) was used to determine the functional annotation clustering using GOTERM_BP/CC/MF_DIRECT, Reactome and Wikipathways. A minimum miRNA/mRNA network was created using miRNet by using differentially expressed miRNAs as seed nodes then the only mRNAs added to the network are those that create links between miRNA seed nodes. This ensures that each mRNA added to the network is targeted by at least two miRNAs.

### 2.10. Statistical analysis

Where expression was compared between two groups, an unpaired Student’s *t*-test was performed and for group comparisons a one-way ANOVA with Tukey’s *post hoc* test was used to determine significance. ANOVA analysis was performed using GraphPad Prism V8 (GraphPad Software, La Jolla, CA, United States). Statistical significance was determined by *p* < 0.05. Relationships between miRNA expression changes and histological measures were tested by Spearman’s correlation using the rcorr function of the Hmisc R package using a loess fit for non-linear correlations (Harrell and Harrell, 2019) and *p* < 0.01 was used as significance threshold. Results from correlation analyzes were graphed with corrplot (Wei et al., 2017).

## 3. Results

### 3.1. Circulating miRNA profiles are altered in response to photo-oxidative damage

Our previous work using the PD model has consistently shown changes in retinal miRNA expression in response to degeneration (Saxena et al., 2015; Chu-Tan et al., 2018; Fernando et al., 2020; Aggio-Bruce et al., 2021; Chu-Tan et al., 2021). In this study we investigated if these changes could also be detected in serum, to investigate the utility of serum miRNAs as a diagnostic for retinal stress, particularly at early stages.

**Figure 1:**
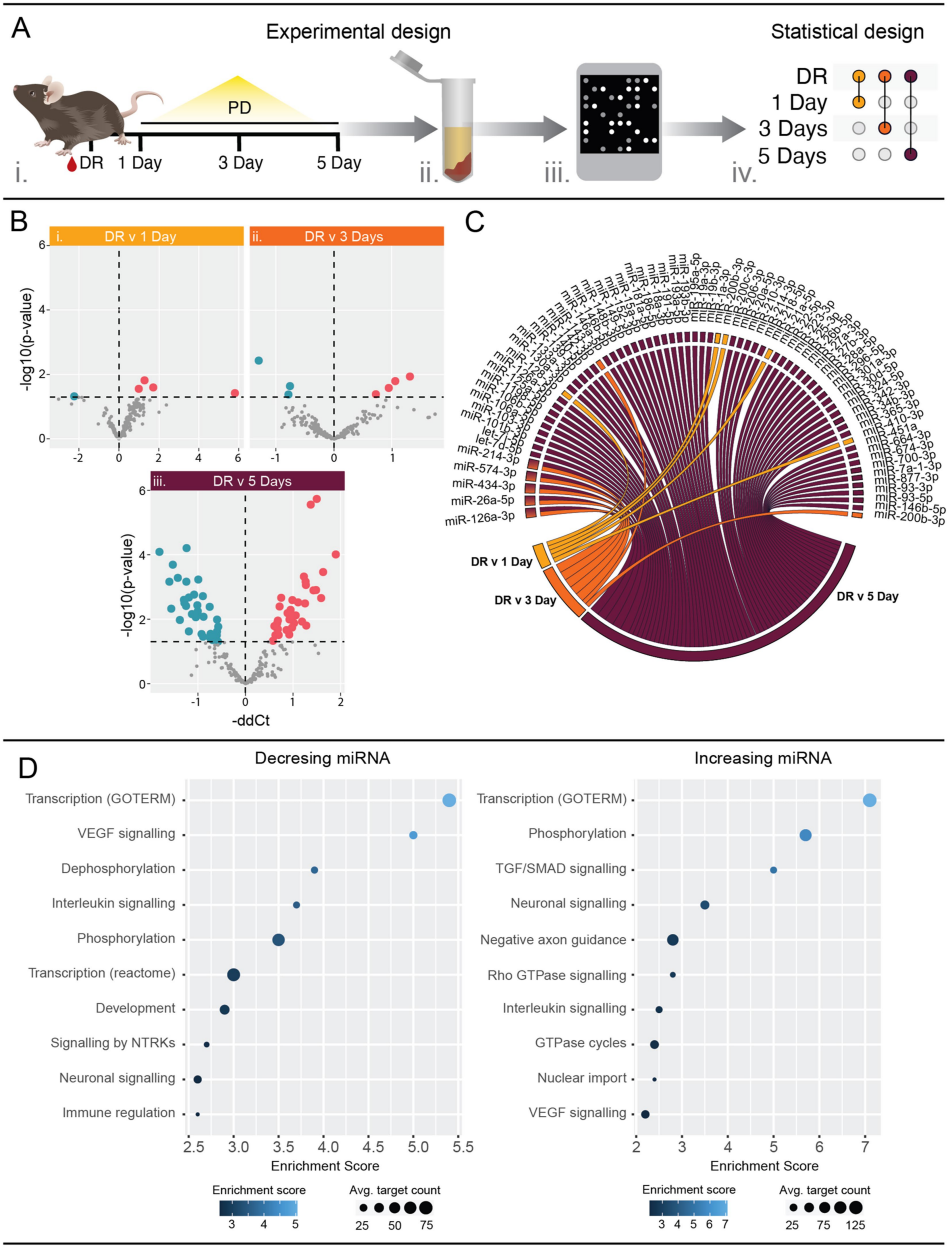
Serum miRNA profiles are altered in response to photo-oxidative damage (PD). **(A)** Schematic showing the experimental workflow. Whole blood was collected from mice at 1, 3 and 5 days PD using dim-reared (DR) animals as control (i). Serum was separated and total RNA purified, enriching for small RNAs (ii). miRNA expression was analyzed using mouse OpenArrays (iii) and differential expression analysis performed between each PD group and the DR control (iv). *n* = 4–6 samples. **(B)** Volcano plots showing differentially expressed miRNAs. Five, seven and 61 miRNAs were significantly (*p* < 0.05) differentially expressed between DR and (i) 1 day PD, (ii) 3 days PD, and (iii) 5 days PD, respectively. See also Table S 2. **(C)** Chord diagram showing overlap of differentially expressed miRNAs across all time points. Five miRNAs (miR-126a-3p, miR-26a-5p, miR-434-3p, miR-574-3p and miR-214-3p) were differentially expressed at both 3 days and 5 days PD. **(D)** Pathway analysis of mRNAs targeted by miRNAs differentially expressed at 5 days PD. Targets were identified using miRNet and filtered for those validated by HITS/PAR-CLIP or reporter assays. Pathway analysis of validated targets was performed using DAVID and significantly enriched (*p* < 0.05) pathways are associated with interleukin, and VEGF and neuronal signaling (see also Supplementary Table S3).

Serum samples were obtained from C57BL/6 J mice at day 0 (DR), 1, 3 and 5 days PD (Figure 1A). A total of five and seven miRNAs were found to be differentially abundant in serum from 1 day PD (Figure 1Bi; Supplementary Table S2A) and 3 days PD (Figure 1Bii; Supplementary Table S2B), respectively. This was markedly increased at 5 days PD with 61 miRNAs differentially abundant (Figure 1Biii; Supplementary Table S2C). Of these, 33 demonstrated a significant increase, with miR-342-3p showing the largest fold increase, while 28 miRNAs exhibited a significant decrease, with miR-20a showing the largest fold change (*p* < 0.05). Further, miRNAs with changing abundance at 1 day did not overlap with those differentially abundant at 3 or 5 days PD (Figure 1C). However, five of the seven miRNAs differentially abundant at 3 days were also differentially abundant at 5 days PD, comprising miR-214-3p, miR-574-3p, miR-434-3p, miR-26a-5p and miR-126a-3p.

Significantly differently abundant serum miRNAs at 5 days PD were submitted to miRNet (Chang et al., 2020) to identify interactions with validated target mRNAs. The target lists were then submitted to DAVID (Huang et al., 2009; Sherman et al., 2022) to identify associations with biological pathways. This revealed a strong association with immune response pathways, VEGF signaling as well as neuronal signaling terms (Figure 1D; Supplementary Table S3).

This data clearly indicates that detectable changes in serum miRNA composition do occur in early stages of retinal damage and are associated with known important molecular pathways involved in AMD. Further, this data revealed that miRNA dysregulation is progressively exacerbated across the PD paradigm, suggesting an association with the underlying pathology.

### 3.2. Circulating miRNA expression patterns correlates to histological changes in the retina

**Figure 2:**
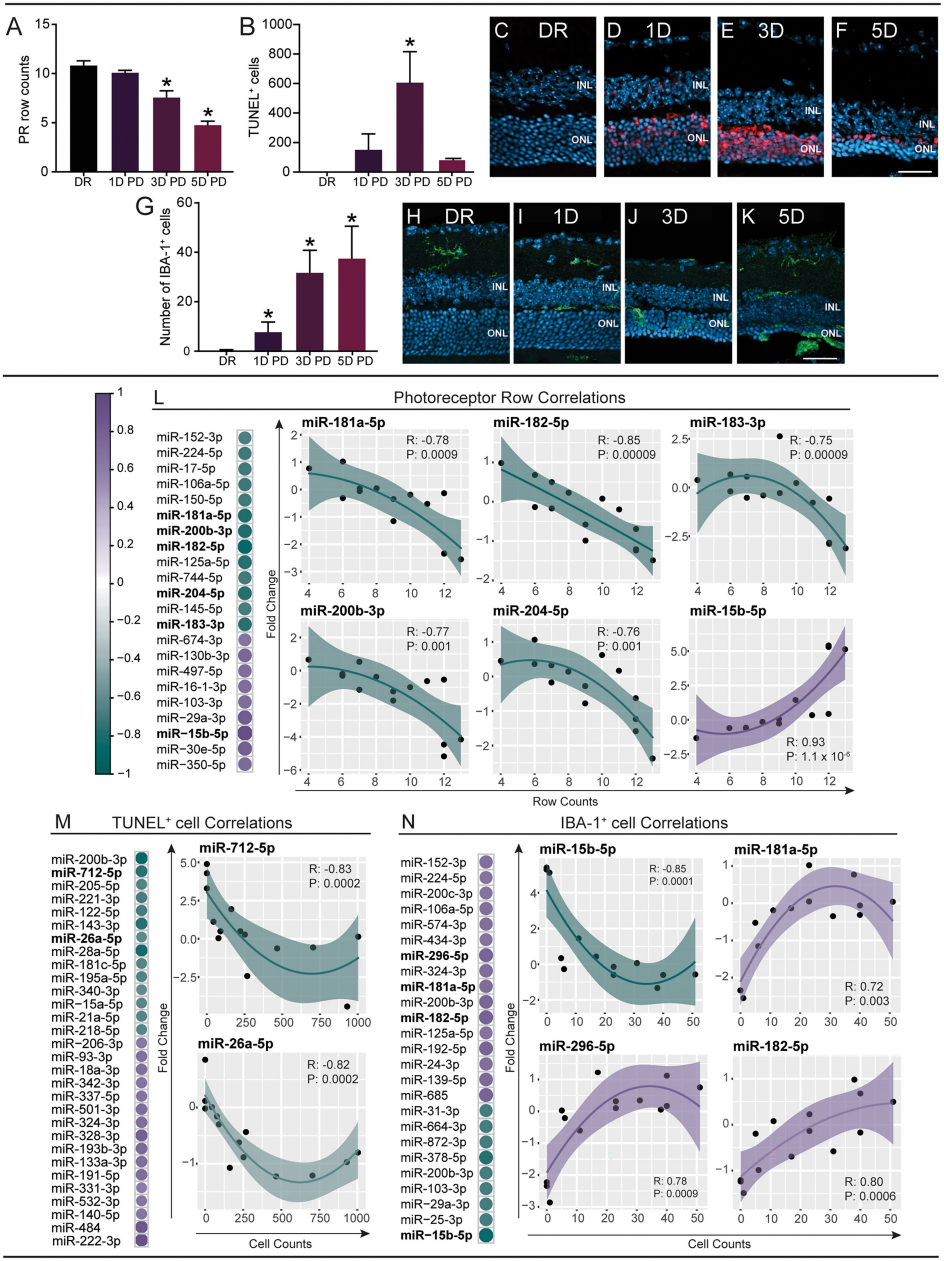
Expression changes of circulating miRNAs correlate to pathological changes in the retina. miRNA expression changes (fold change) at each PD time point were compared to photoreceptor row counts (PR rows), TUNEL^+^ cell counts and IBA^+^ cell counts using Spearman’s correlation (*p* < 0.01; see Supplementary Table S4). **(A–K)** Immunohistochemical analysis of retinal cryosections. **(A–F)** Apoptotic photoreceptor cells were stained using TUNEL and sections were counterstained with bisbenzimide [representative images **(C–F)**]. There was a decrease in the number of PR rows at the site of damage (500 μm from the optic nerve head) across the PD paradigm, which was significant at 3 days and 5 days PD **(A)**. Photoreceptor cell death increased at 1 day PD and was significantly increased at 3 days PD, before decreasing at 5 days PD **(B)**. **(G–K)** Inflammation (infiltrating microglia/macrophages) was analyzed by immunostaining cryosections using an anti-IBA-1 antibody and counting IBA-1^+^ cells in the ONL [Representative images of the site of damage **(H–K)**]. A significant increase was identified at 1 day, 3 days and 5 days PD **(G)**. Scale bar = 500 μm. INL = inner nuclear layer, ONL = outer nuclear layer. Error bars indicate SEM. Statistical significance was determined by one-way ANOVA (*n* = 12–16 per group, *p* < 0.05). **(L)** The expression changes of 22 miRNAs were significantly correlated to PR row counts. **(M)** The expression changes of 30 miRNAs were significantly correlated to TUNEL^+^ cell counts. **(N)** 25 miRNAs were significantly correlated to IBA-1^+^ cell count. The relationship between miRNA fold changes and PR, IBA, TUNEL was summarized using a loess fit. The fit clearly show that most relationships are non-linear therefore we used Spearman’s rank-based statistic to examine the significance of the correlation. Shaded area shows the 95% confidence interval.

To understand whether the progressive serum miRNA changes are indeed indicative of the underlying retinal pathology, we investigated if changes in miRNA abundance could be correlated to specific histological measures, including thinning of the ONL (photoreceptor row count; Figure 2A), apoptosis (TUNEL^+^ cell count; Figures 2B–F) and recruitment of immune cells into the outer retina (IBA-1^+^ cell count; Figures 2G–K). The individual fold change per sample of all miRNAs was plotted against these histological measures and Spearman’s correlation analysis was performed to identify miRNAs with significant correlation (*p* < 0.05; Supplementary Table S4; Supplementary Figure S2). Due to the large number of significant findings, only correlations with *p* < 0.01 are shown here. Photoreceptor row counts were found to correlate to 22 miRNAs (Figure 2L), with IBA-1^+^ cell counts correlating to 25 miRNAs (Figure 2M) and TUNEL^+^ cell counts showing significant correlation to 30 miRNAs (Figure 2N), which included miRNAs with known roles in the retina (shown in individual plots) such as miR-182, miR-182 and miR-26a.

Taken together, we show distinct correlations between serum miRNA changes and changes in retinal morphology, strongly suggesting that miRNAs are reflective of pathology. This highlights the need for further exploration into serum miRNA modulations in relation to the progression of retinal degeneration.

### 3.3. Circulating miRNAs are differentially accumulated in patients with reticular pseudodrusen and geographic atrophy

**Figure 3:**
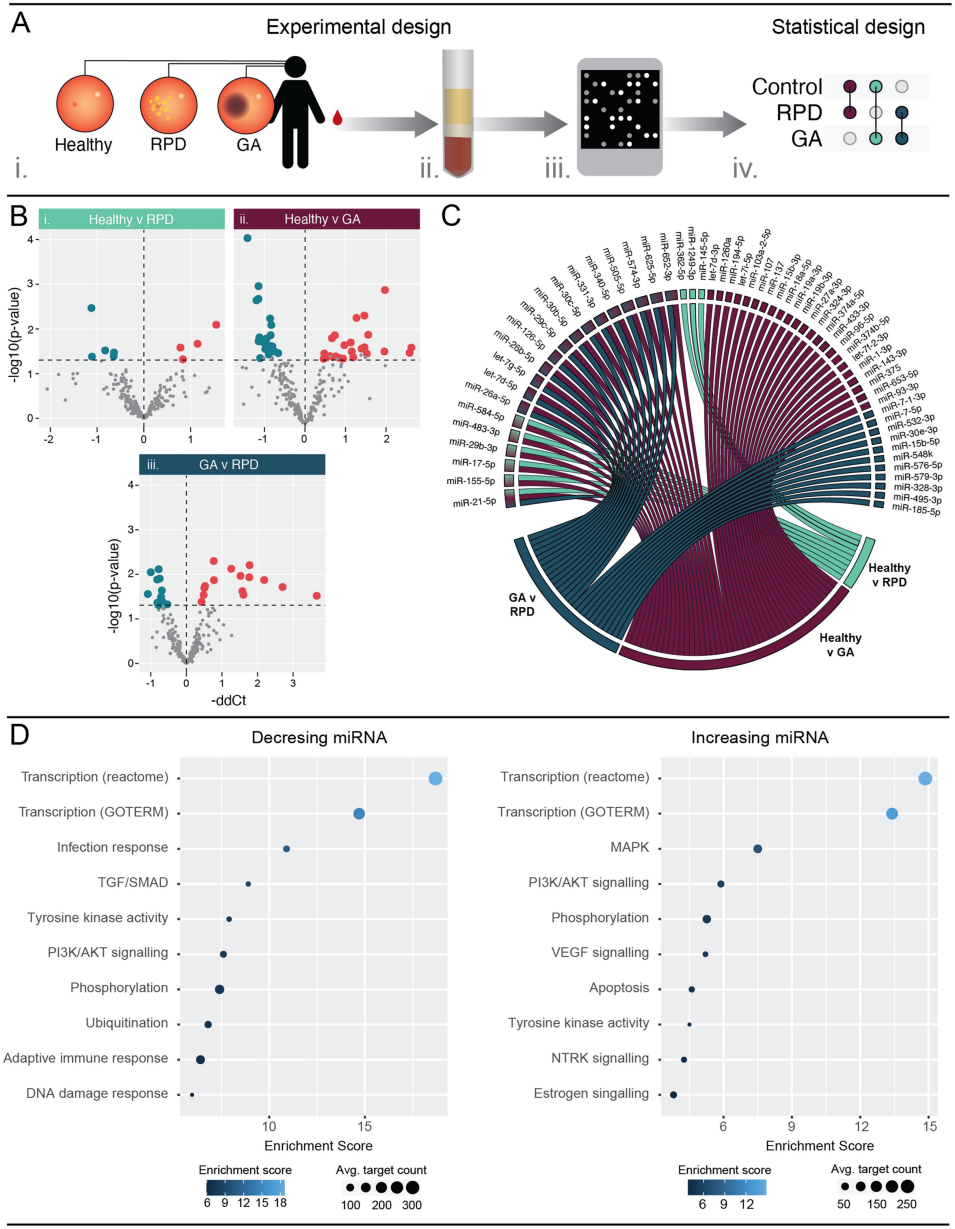
Circulating serum miRNA are differently expressed in patients with RPD and GA when compared to healthy controls. **(A)** Schematic showing the experimental workflow. Blood samples were obtained from patients with no retinal pathology (healthy), reticular pseudodrusen (RPD), or atrophic AMD (GA) (i). Serum was separated and RNA purified enriching for small RNAs (ii). miRNA expression was analyzed using human OpenArrays (iii) and differential expression analysis performed between healthy and disease groups (iv) (*n* = 5–10 samples). **(B)** Volcano plots showing differentially regulated miRNAs. Nine and 43 miRNAs were significantly (*p* < 0.05) modulated in RPD and GA patients compared to healthy controls. Additionally, 26 miRNAs showed significant modulation between RPD and GA groups. See also Table S5. **(C)** Chord diagram showing overlap of modulated miRNAs across all groups. A single miRNA, miR-21-5p, was significantly regulated between all three groups. Five miRNAs were differentially regulated in both GA and RPD groups compared to controls, and 14 miRNAs were significantly regulated in GA patients compared to both healthy and RPD groups. **(D)** Pathway analysis of mRNAs targeted by miRNAs significantly modulated in GA patients were identified using miRNet and filtered for those validated by HITS/PAR-CLIP or reporter assays. Pathway analysis of validated targets was performed using DAVID and significantly enriched (*p* < 0.05) pathways are associated with transcription, protein activity and PI3K/AKT signaling. MAPK and apoptosis signaling was uniquely associated with mRNA targets of upregulated miRNAs, whereas infection and adaptive immune response were uniquely associated with mRNA targets of downregulated miRNAs. See also Supplementary Table S6.

Serum samples were obtained from healthy individuals and patients diagnosed with reticular pseudodrusen (RPD) and geographic atrophy (GA) from the Lions Eye Institute (WA, Australia) and analyzed for relative miRNA levels (Figure 3A). We identified a significant difference in the abundance of nine miRNAs between healthy controls and RPD patients, with four upregulated and five downregulated (Figure 3Bi; Supplementary Table S5A; *p* < 0.05). Comparatively, there were a total of 43 miRNAs that were significantly differentially abundant between healthy controls and GA patients (Figure 3Bii; Supplementary Table S5B). Of these, 20 miRNAs were downregulated and 23 were upregulated. Comparison between RPD and GA patients revealed differential abundance of 26 miRNAs, with 11 downregulated and 15 upregulated in GA (Figure 3Biii; Supplementary Table S5C).

Several miRNAs were modulated in multiple groups (Figure 3C). Five miRNAs, miR-584-5p, miR-483-3p, miR-29b-3p, miR-17-5p and miR-155-5p, were modulated in both the healthy-GA and healthy-RPD comparisons, whereas 14 miRNAs were modulated in both the healthy-GA and RPD-GA comparisons. Interestingly, one miRNA, miR-21-5p, was found to be modulated in all three comparisons, with a-0.63 and-1.41 fold change in RPD and GA, respectively (Figure 3C; Supplementary Table S5).

To determine the biological pathways these differentially abundant miRNAs might play a role in, we performed pathway analysis using DAVID. As the number of differentially regulated miRNAs in RPD patients was insufficient for pathway analysis, we focussed on those dysregulated in GA. Significantly modulated serum miRNAs were submitted to miRNet (Chang et al., 2020) to identify interactions with validated target mRNAs before pathway analysis was performed. Enriched processes involved in transcription and protein activity were common to mRNA targets of both upregulated and downregulated miRNAs (Figure 3D; Supplementary Table S6). Pathways involved in infection response, TGF/SMAD signaling, and adaptive immune response were associated with mRNA targets of downregulated miRNAs, while MAPK, VEGF, NTRK and apoptosis signaling were associated with mRNA targets of upregulated miRNAs. Taken together, these findings aligned to those observed in the PD mouse model.

### 3.4. Modulation of miRNAs across patients correlates to retinal volume

**Figure 4:**
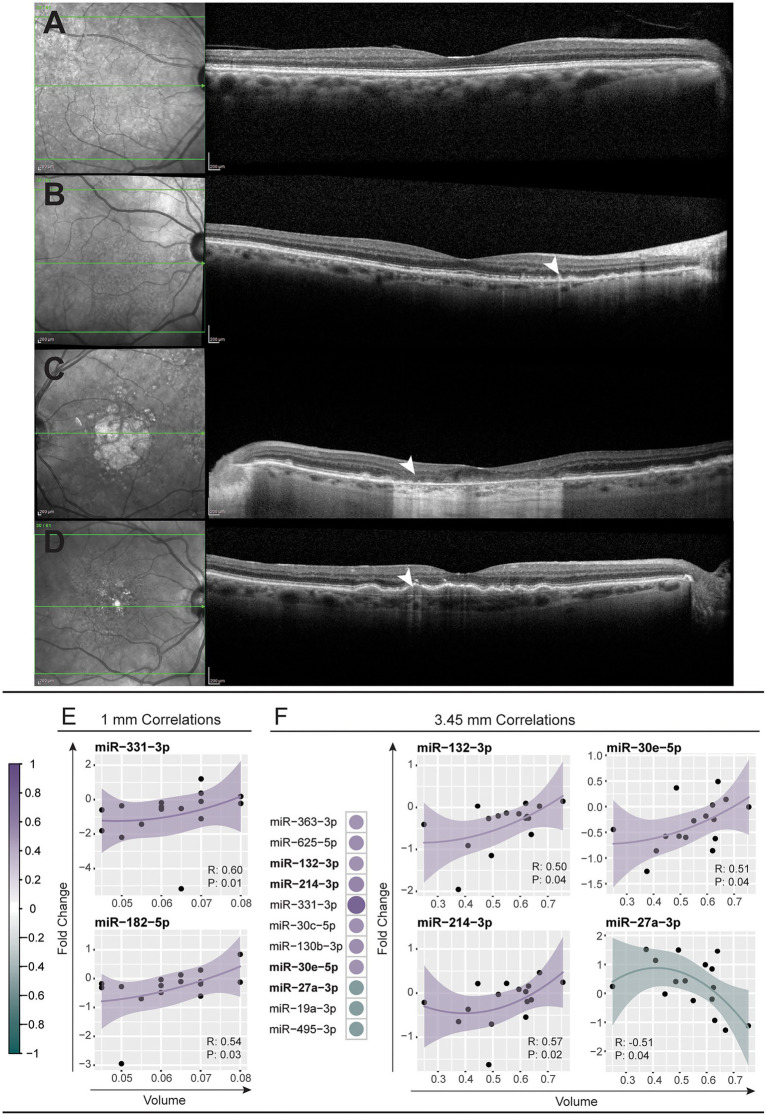
Modulation of miRNAs across patients corelates to retinal volume. **(A–D)** Representative near-infrared (left column) and optical coherence tomography (right column) fundus images across the macular region of a healthy control **(A)** and patients with reticular pseudodrusen **(B)**, geographic atrophy secondary to AMD **(C)**, and large confluent drusen with early RPE depigmentation and hypertransmission defect **(D)**. **(E,F)** Modulations in miRNA abundance across all patient samples were correlated (Spearman’s correlation, *p* < 0.05) against the volume of the central retina within 1 mm and 3.45 mm diameter circles centered on the macula (see also Supplementary Table S7). **(E)** Two miRNAs, miR-331-3p and miR-182-5p, showed positive correlation to the 1 mm volume of patient retinas. **(F)** Eleven miRNAs correlated to the 3.45 mm volume with scatter plots shown for the top four most correlated miRNAs. A positive correlation was observed for miR-30e-5p, miR-132-3p and miR-214-3p and a negative correlation for miR-27a-3p. The solid line indicates perfect correlation with the shaded area indicating variance.

As we were able to correlate changes in serum miRNA abundance to retinal morphology in the PD model, we asked whether serum miRNA changes are also indicative of patient retinal pathology. Representative fundus and optical coherence tomography (OCT) images demonstrated distinct retinal pathology between patients with drusen, RPD and atrophy/thinning of the outer retina (Figures 4A–D; Supplementary Table S7). The volume of the outer nuclear layer (ONL), at circle diameters; 1, 2.22, 3.45 mm centered around the macula, was measured using the Heidelberg spectralis software and correlated to miRNA expression (Supplementary Figure S3). The abundance changes of two miRNAs, miR-331-3p and miR-182-5p, were found to negatively correlate to the 1 mm diameter volume of the ONL (Figure 4E). The changes in abundance of 11 miRNAs correlated to the total volume of the ONL measured (3.45 mm diameter), three of which demonstrated a positive correlation (Figure 4F). This data demonstrates the potential for this type of analysis to be utilized as a strategy for predicting complex disease outcomes from serum miRNA abundance. However, future investigation using a larger sample size is required.

### 3.5. Modulated serum miRNAs show similarities between 5 days PD and patients diagnosed with geographic atrophy

**Figure 5:**
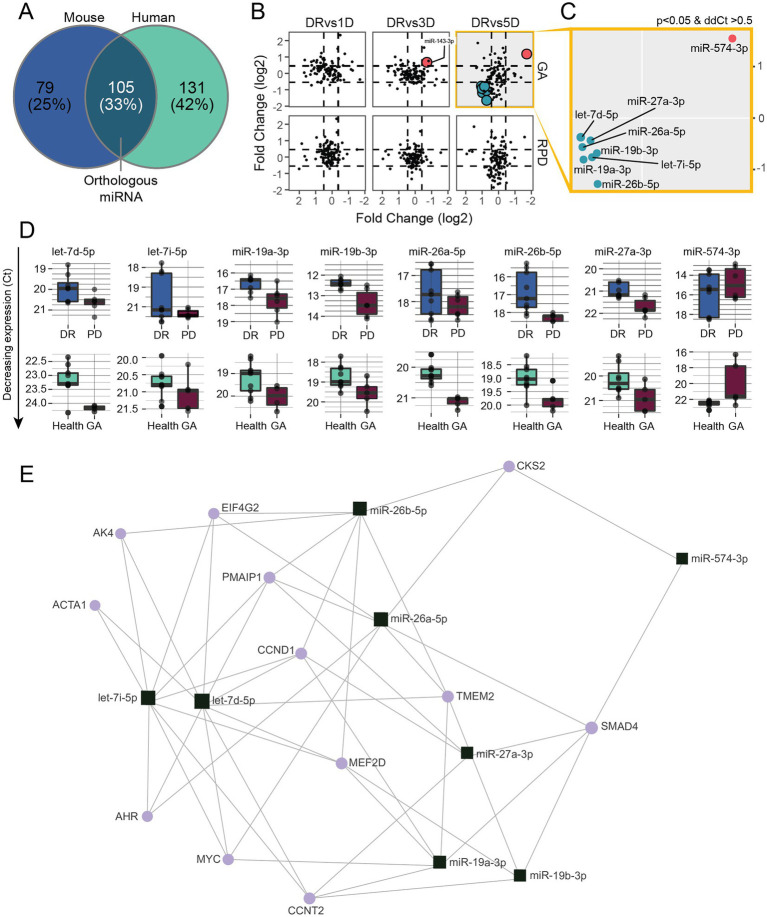
Modulated serum miRNAs show similarities between 5 days PD and human GA patients. **(A)** Venn diagram showing the overlap of miRNAs detected in mouse and human samples. **(B)** miRNA expression changes (ddCt) at 1, 3 and 5 days PD were plotted against miRNA expression changes (ddCt) of RPD and GA patients. **(C)** Eight miRNAs were significantly (*p* < 0.05) differentially regulated in both 5 days PD and GA samples, with miR-574-3p upregulated and miR-27a-3p, miR-26a-5p, miR-26b-5p, miR-19a-3p, mi-19b-3p let-7d-5p and let-7i-5p downregulated in both groups. **(D)** Box plots showing the expression changes of each miRNA differentially regulated in both groups. **(E)** Minimum miRNA/mRNA network analysis of the eight common differentially regulated miRNAs using miRNet. The plot shows the predicted interactions of each miRNA (square) with several key genes (circles) involved in transcription (CCNT2, SMAD4), inflammation (AK4, AHR) and apoptosis (PMAIP1, MEF2D).

Although human and mouse gene expression profiles are distinct, our data clearly indicates similarities; not unexpected as the PD model recapitulates key aspects of AMD. Therefore, we asked whether using miRNAs that are dysregulated in both PD and human patients could be used to define a miRNA AMD disease signature. Firstly, we compared all miRNAs present in both datasets and identified 105 orthologous miRNAs (Figure 5A). Next, we plotted the differential abundance of all 105 human and mouse miRNAs, which revealed commonly modulated miRNAs (eight) in the 5-day PD and GA samples (Figure 5B). One miRNA, miR-574-3p, was significantly upregulated and seven miRNAs were significantly downregulated in both samples (Figures 5C,D). Minimum network analysis using miRNet revealed interactions with genes involved in transcription, inflammation and apoptosis (Figure 5E; Supplementary Table S6), strongly suggesting that these miRNAs are indicative of retinal pathology.

## 4. Discussion

Due to the central role of miRNAs in the regulation of biological processes, especially inflammation and disease progression (He and Hannon, 2004), circulating miRNA have been identified as an attractive, non-invasive prognostic tool for multiple neurological disorders (Li et al., 2007; Chen et al., 2008; Mitchell et al., 2008; Jung et al., 2010; McDonald et al., 2011; Weiland et al., 2012), including AMD (Grasso et al., 2014). However, candidate miRNA diagnostic markers of AMD have primarily been established based on late stages of disease, with little investigations into early-stage disease markers (Ertekin et al., 2014; Grassmann et al., 2014; Szemraj et al., 2015; Ménard et al., 2016; Berber et al., 2017; Ren et al., 2017; Romano et al., 2017; Szemraj et al., 2017; ElShelmani et al., 2020). Further, to our knowledge, existing studies have not gone beyond simple differential expression profiling to narrow the target panel. This study advances AMD biomarker research in multiple ways: (1) utilizing a pre-clinical animal model that recapitulates aspects of AMD as well as human patient samples, (2) identification of modulated miRNAs that correlated with retinal pathology, and (3) overlapping pre-clinical and clinical data to refine a preliminary miRNA panel with higher stringency.

Here we demonstrate that circulating miRNAs are modulated in response to photo-oxidative damage-induced photoreceptor degeneration and immune cell migration, even at early-stages. Furthermore, *via* pathway analysis, we showed that potential diagnostic miRNA targets demonstrate an association with neuronal processes and inflammation. Additionally, we show that the modulation of some miRNAs correlated to distinct changes in retinal morphology. We then corroborated these findings using human serum from patients with a diagnosis of RPD or GA. Finally, we identified candidate miRNAs that showed similar modulations between human and rodent samples at late stages. Taken together, this work identified circulating miRNA changes that closely reflect pathogenic processes in the retina, making them ideal candidate biomarkers for early-stage AMD diagnostics.

### 4.1. Circulating miRNAs are linked to photoreceptor degeneration

The clinical potential of diagnostic biomarkers relies not only on the ability to accurately detect the presence of pathological features, but, more importantly, the identification of disease onset prior to the development of pathologies (drusen deposits and pigmentary changes) through traditionally imaging techniques (fundus imaging) (Klein et al., 1992, 2007; Fine et al., 2000; van Lookeren et al., 2014). The regulation of miRNAs in late-stage AMD has been widely reported (Ertekin et al., 2014; Grassmann et al., 2014; Szemraj et al., 2015; Ménard et al., 2016; Berber et al., 2017; Ren et al., 2017; Romano et al., 2017; Szemraj et al., 2017; ElShelmani et al., 2020) however, with minimal overlap between studies, likely due to compounding factors such as co-morbidities, sample collection, and method of analysis.

Given the limitations of early diagnostic capabilities, we utilized a well-established rodent PD model that recapitulates important facets of AMD (Natoli et al., 2016) to identify differentially modulated miRNA at all stages of disease progression. We showed here that, although early stages of photo-oxidative damage (1-day) do not show substantial histological changes, there are already detectable miRNA changes in the circulation. These miRNAs included miR-128a-3p, miR-200b-3p, miR-410-3p and miR-218-5p, which have shown to be involved in inflammation and oxidative stress processes and to play a role in neurodegenerative disorders including AMD (Wang et al., 2019; Wu et al., 2020; Xiong et al., 2021; Yang et al., 2021; ElShelmani et al., 2021a; Acuña et al., 2022). This suggests that their presence in circulation reflects early changes in retinal homeostasis and we hypothesize that these miRNAs have the potential to be early indicators of disease onset. Importantly, we show that modulation of these four miRNAs was unique to 1 day PD, demonstrating their utility as a signature for early degeneration.

Of further interest was miR-126a-3p, which increased in serum at 3 days and 5 days PD in correlation to changes in photoreceptor rows and IBA-1^+^ cell counts. MiR-126a-3p has been shown to protect against oxidative stress in the brain (Tan et al., 2021), be associated with protection of photoreceptors in retinitis pigmentosa (Wang and Smith, 2019), and to decrease in the choroid in response to neovascularisation (Desjarlais et al., 2019).

We show that in response to the whole PD time-course, molecular pathways linked to modulated circulating miRNAs are associated with Interleukin signaling, cell migration and TGF/SMAD signaling. This is unsurprising, as PD-induced miRNA modulations in the retina are primarily associated with the inflammatory response and related pathways (Saxena et al., 2015). Further, signaling between the retina and circulation under ongoing stress is essential to mediate the infiltration of monocytes into the retina during degeneration (Perez and Caspi, 2015; Zhou et al., 2017). Of note, in this study we found that a number of modulated miRNAs correlated to IBA-1^+^ cell counts, such as miR-148a-3p and miR-152-3p, which have been shown to play a role in the inflammatory response in both the retina/choroid (Tsunekawa et al., 2017; Desjarlais et al., 2019) and circulating peripheral blood mononuclear cells (Wang et al., 2020; Zhang J et al., 2020; Tao et al., 2021). Additionally, miR-148a-3p has been shown to promote the classical M1 activation of macrophages, thereby increasing pro-inflammatory properties of the cell (Huang et al., 2017; Zhang P et al., 2020). Conversely, miRNAs miR-103, miR-181a and miR-24, which are also correlated to IBA-1^+^ cell counts, have been shown to regulate pro-inflammatory factors expressed by microglia (Bian et al., 2020). This regulation has been shown to occur *via* communication by extracellular vesicles (EVs) from neural progenitor cells to microglia. Further, it has been suggested that EVs released from photoreceptors during early stages of retinal degeneration communicate a stress response within the retina (Wooff et al., 2020). Therefore, it is possible that these EVs enter the blood stream from the retina contributing to the modulations in miRNA abundance identified. Further, several studies have shown that EV-based miRNA biomarker development could result in a more specific and stringent panel for disease detection (Cheng et al., 2014; Barile and Vassalli, 2017), highlighting the need of future work to investigate the modulations of serum EV-derived miRNAs in AMD.

Taken together, these findings support the notion that these circulating miRNA are associated with retina-specific degeneration, leading to the identification of prospective diagnostic miRNAs.

### 4.2. Clinical serum miRNA profiles demonstrate consistencies with mouse data and current literature

Existing clinical studies into miRNAs as biomarkers for AMD, both atrophic and neovascular AMD, have identified miR-27a, miR-34, miR-106b, miR-126, miR-146a, miR-155, miR-361, miR-424-5p, miR-93-3p, and miR-21-5p among many others (Ertekin et al., 2014; Grassmann et al., 2014; Szemraj et al., 2015; Ménard et al., 2016; Ren et al., 2017; Romano et al., 2017). Our investigation of clinical samples revealed miR-93-3p to increase in atrophic AMD patient samples. This increase potentially aligns to current findings by ElShelmani et al. (2020), that identified an increase in miR-93 in the serum of neovascular AMD patients (ElShelmani et al., 2020), however, did not define which arm of miR-93 (−3p or-5p), a drawback common to existing AMD biomarker literature. As each miRNA exhibits unique biological functions, identifying the precise sequence is essential for future functional studies.

To further validate our findings, we correlated the human serum miRNA modulations to a histological measure, ONL volume. We found correlations between the abundance of three miRNA, miR-19a, miR-27a and miR-132-3p, showing a trend direction that aligns to previous findings in AMD patients (Ren et al., 2017; Romano et al., 2017; Elbay et al., 2019; ElShelmani et al., 2020, 2021b). To further refine our panel to be highly stringent, we identified differentially regulated miRNAs common to the PD model and AMD patient samples, which revealed that let-7i-5p, let-7d-5p, miR-19a-3p, miR-19b-3p, miR-26a-5p, miR-26b-5p, miR-27a-3p, and mir-574-3p are similarly modulated. Some of these miRNAs have been identified previously in association with AMD, with miR-26a-5p and miR-27a-3p showing directional changes consistent with the literature (Grassmann et al., 2014; Szemraj et al., 2015) and let-7d-5p and miR-574-3p showing a directional change opposite to that reported in the literature (Ertekin et al., 2014; ElShelmani et al., 2020). Additionally, miR-27a-3p is a mediator of the inflammatory response (Zhang et al., 2018), and its overexpression was associated with increased oxidative stress and retinal damage (Ren et al., 2021), and increasing in aging mouse retinas (Hermenean et al., 2021). Interestingly, here we show that modulation in miR-26a-5p and miR-27a-3p correlated with histological features, specifically TUNEL^+^ cell count and retinal volume, respectively.

Importantly, we further identified several modulated serum miRNAs that are known to be highly abundant in the human retina, including miR-96, miR-26a/b and miR-143-3p interestingly, these miRNAs have not been previously reported to be differentially regulated in circulation in response to AMD. Of note was miR-96, a member of the miR-182/96/183 miRNA cluster, which is highly expressed in mature photoreceptors and the inner nuclear layer of the retina (Pawlick et al., 2021). Similarly, the mouse serum data revealed a change in miR-181a, which is highly expressed in the retina, particularly photoreceptors (Pawlick et al., 2021). Consistent with these functions, the mouse data demonstrated a correlation of miR-181a, miR-182 and miR-183 to photoreceptor row counts. To our knowledge this cluster has not previously been identified in serum in association with AMD.

Taken together, these findings support our hypothesis that changes in retinal homeostasis can be identified in the circulation, as these miRNAs are likely to originate from the retina. However, in the current study, we identified a number of miRNAs that showed modulations that were different from previous reports. For instance, here miR-21-5p was shown to decrease in both RPD and atrophic AMD patients, whereas miR-21-5p was previously identified to increase in patients with neovascular AMD (Szemraj et al., 2015). Similar to Ren et al. (2017), we identified significant modulations of miR-29b in RPD and GA serum samples, and of miR-27a-3p in GA alone. However, in opposition to Ren et al. (2017), we observed downregulation of both in our data. Moreover, our study showed inconsistency with multiple studies that have reported miR-223, miR-146, and/or miR-155 to be promising biomarkers for retinal degeneration (Ertekin et al., 2014; Szemraj et al., 2015; Romano et al., 2017; ElShelmani et al., 2020). There are several pre-analytical and post-analytical factors that could influence these discrepancies (McDonald et al., 2011). Firstly, our small sample size, sample collection, processing, and quality control could influence the miRNA profile (Blondal et al., 2013; Keller et al., 2017; Lee et al., 2017). The findings reported by Ren et al. (2017) and Ertekin et al. (2014) analyzed whole blood and plasma, respectively, whereas we profiled serum fractions to avoid miRNA contributions from cellular components. Further, we used an OpenArray™ approach, which only targets a subset of known miRNAs and is an amplification-based technique. As such, the normalization strategies are considered to have a significant outcome on results (Faraldi et al., 2019). Here we used a set of normalization controls based on global normalization, one of the most commonly used and recommended normalization tools (Mestdagh et al., 2009). We further used highly stringent thresholds for our amplification data to minimize the chance of a type I error. Additionally, we analyzed the contribution of covariates such as sex and age to miRNA expression and corrected our analysis accordingly, which is rarely reported in the existing literature. Other known AMD risk factors such as diet and smoking and genetic predisposition should be taken into account in future studies. Further, variability due to normalization can be avoided by measuring absolute abundance using sequencing techniques, which would also allow unbiased detection of all miRNAs present. Additionally, sequencing approaches would enable transcriptome-wide analyzes, which could include other small RNA species as well as non-coding RNAs and mRNAs, facilitating the development of a more specific diagnostic panel.

Overall, while we have utilized a number of strategies to identify and validate miRNA targets, there remains a clear need for further optimization and expansion of this body of work to a larger cohort.

### 4.3. Translation to a clinical setting through targeted analysis strategies mediated by biosensors

While the development of an accurate predictive panel for AMD pathogenesis is still in its infancy, there remains an unmet need for the development of tools that can allow effective, specific and timely detection of miRNAs in a clinical setting. The successful implementation of a biomarker panel to support clinical diagnosis relies on fast and robust detection of molecules of interest. Using sequencing-based methods are costly and require skilled personnel, an impediment to translation into clinical use. Target-specific methods such as electrochemical detection through biosensors present ideal strategies for translation into the clinic. Biosensors do not require special training and can be miniaturized, ideal for point-of-care or at home utilization. Thus, biosensors are excellent choices as companion diagnostics for rapid diagnosis as well as ongoing day-to-day monitoring of therapeutic intervention success. A wide variety of studies have manufactured electrochemical biosensors for miRNA detection (Gillespie et al., 2018), but their direct application in serum samples remains challenging, highlighting the need for further research.

### 4.4. Conclusion

The manifestation and progression of atrophic AMD in its early stages is relatively asymptomatic, and once vision loss ensues there is no effective treatment available. Therefore, early, minimally invasive detection is essential for the development of novel interventions. However, the nature of late clinical diagnosis and the presence of co-morbidities creates a challenge in identifying early markers of disease. By utilizing the rodent photo-oxidative damage model we were able to identify miRNA, such as let-7i/g-5p, miR-26a-5p, miR-19a-3p and miR-574-3p, in circulation indicative of early stages of neuronal cell death and subsequent retinal inflammation. We were further able to show similar abundance changes of these miRNAs in human AMD samples, highlighting their potential as indicators of late-stage AMD. Finally, quantification of the key miRNAs presented here in a larger human patient cohort will provide essential validation in the progression of this panel to a clinical setting. This analysis demonstrates a unique methodological approach to elucidate a miRNA diagnostic signature of both early and late AMD.

## Data availability statement

The original contributions presented in the study are included in the article/Supplementary material, further inquiries can be directed to the corresponding author.

## Ethics statement

The studies involving human participants were reviewed and approved by University of Western Australia Human Research Ethics Committee. The patients/participants provided their written informed consent to participate in this study. The animal study was reviewed and approved by Australian National University Animal Experimentation Ethics Committee.

## Author contributions

RA-B conducted PD experiments including sample processing and running the associated OpenArrays™. RA-B, US, and SD performed human sample processing including running on the OpenArrays™. FC, SM, and RH provided the human blood samples and collected and graded the retinal images. RA-B and AC performed the OpenArray™ data analysis. RA-B and US were major contributors to writing the manuscript. RN and FC conceived the study. RN provided funding to conduct the study. All authors have read and approved the final manuscript.

## Funding

The authors acknowledge the Discovery Translation Fund (RN, RAG), the ANU Translational Fellowship (RN), the NHMRC Ideas grant (APP1127705; RN), the NSW RNA Future Leader Program (EMC Researcher Grant; US) and the NHMRC Investigator Grant (MRF1142962; FC) for support of this work.

## Conflict of interest

The authors declare that the research was conducted in the absence of any commercial or financial relationships that could be construed as a potential conflict of interest.

## Publisher’s note

All claims expressed in this article are solely those of the authors and do not necessarily represent those of their affiliated organizations, or those of the publisher, the editors and the reviewers. Any product that may be evaluated in this article, or claim that may be made by its manufacturer, is not guaranteed or endorsed by the publisher.

## Abbreviations

AMD: age related macular degeneration
ARVO: association for research in vision and ophthalmology
ANU: Australian National University
DR: dim-reared
GA: geographic atrophy
IBA-1: ionized calcium-binding adaptor molecule 1
INL: inner nuclear layer
miRNA: microRNA
ONL: outer nuclear layer
PD: photo-oxidative damage
RPD: reticular pseudodrusen
TUNEL: terminal deoxynucleotidyl transferase dUTP nick end labeling
