# Supplementary material for: Serum miRNA modulations indicate changes in retinal morphology
Source: Front Mol Neurosci. 2023 Mar 3;16:1130249. doi: 10.3389/fnmol.2023.1130249 (PMC10020626; doi:10.3389/fnmol.2023.1130249)
Supplement: Supplementary file 1 [file Data_Sheet_1.docx]

**Supplementary Material**

**Figure S1**

**(A)** Distribution of Ct data before (top) and after (bottom) filtering. Samples were filtered using an amplification score < 1.24 and Ct value > 30. Samples that were outside the top/bottom 10% quantile of each group were also filtered. **(B)** Boxplots of Ct distribution of patient samples before (left) and after (right) normalisation using rank invariant miRNAs as reference genes. **(C)** PCA plots demonstrating the clustering of samples based on covariates (experimental batch (Array), sex, age). **(D)** The effect of each covariate on the distribution of p-values.

**Figure S2**

miRNA expression changes (fold change) at each PD time point were correlated to **(A)** photoreceptor row counts (PR rows) and IBA^+^ cell counts and **(B)** TUNEL^+^ cell count using Spearman’s correlation analysis.

**Figure S3**

Modulations in miRNA abundance across all patient samples were correlated (Spearman’s correlation test, p < 0.05) against the volume of the central retina within 1 mm (macula) and 3.45 mm (total) diameter circles centred on the macula (see also Table S7).

**Table S1**

Summary of patient details of patients recruited into this study.

**Table S2**

Significant miRNA changes during **(A)** 1 day, **(B)** 3 days, and **(C)** 5 days of photo-oxidative damage compared to the dim-reared control.

**Table S3**

MirNet Pathway analysis of mRNAs targeted by miRNAs **(A)** downregulated and **(B)** upregulated at 5 days PD.

**Table S4**

Spearman’s correlation analysis between modulated miRNAs and mouse retinal histological measures.

**Table S5**

Significant miRNA changes in **(A)** RPD patients vs healthy controls, **(B)** GA patients vs healthy controls, and **(C)** GA patients vs RPD patients.

**Table S6**

MirNet Pathway analysis of mRNAs targeted by miRNAs **(A)** downregulated and **(B)** upregulated in GA patients.

**Table S7**

Spearman’s correlation between modulated miRNAs and human retinal histological measures.
