# Supplementary figures and images for: Serum miRNA modulations indicate changes in retinal morphology
Source: Front Mol Neurosci. 2023 Mar 3;16:1130249. doi: 10.3389/fnmol.2023.1130249 (PMC10020626; doi:10.3389/fnmol.2023.1130249)

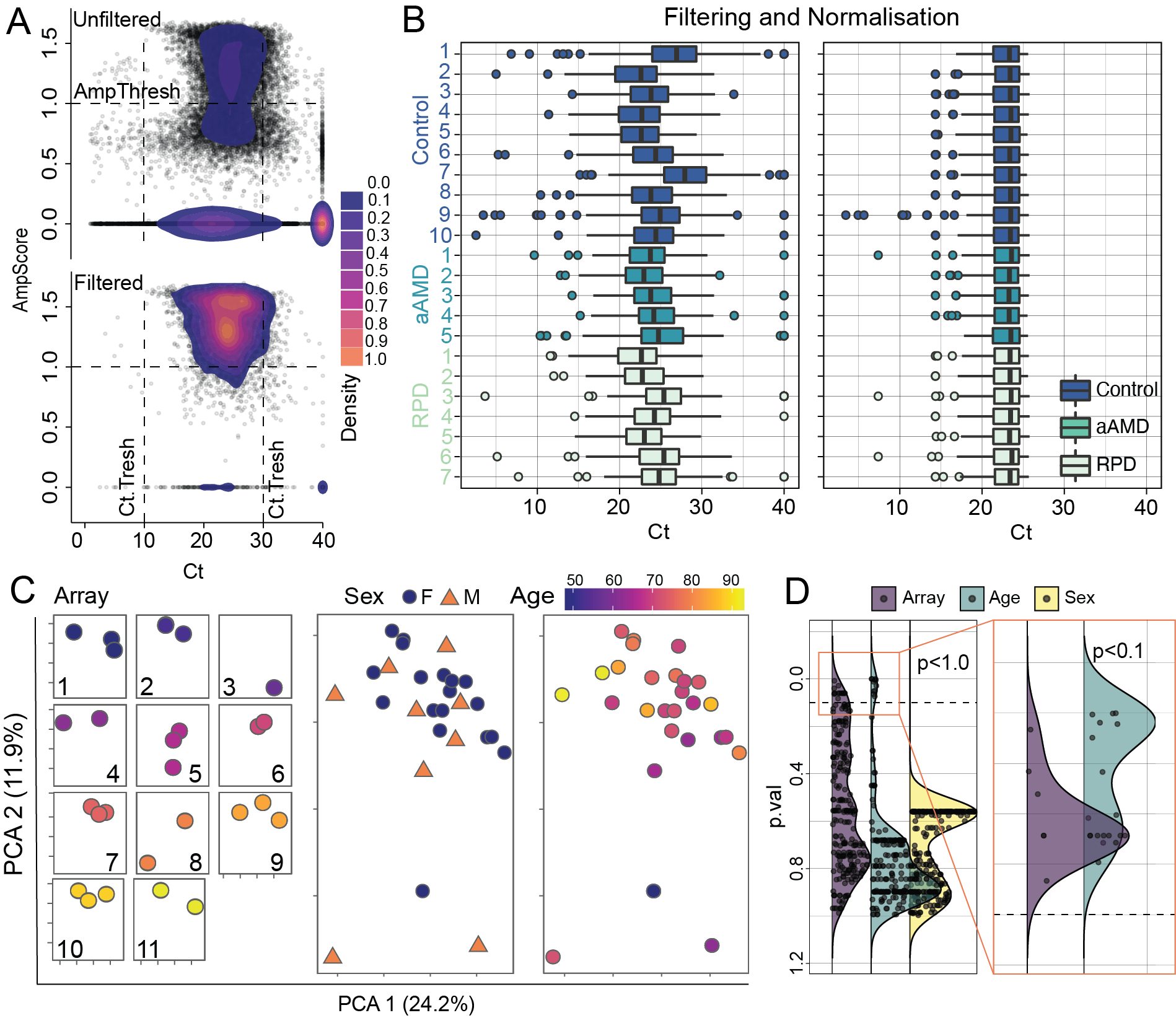

Supplement: Supplementary file 2 [file Image_1.PNG]

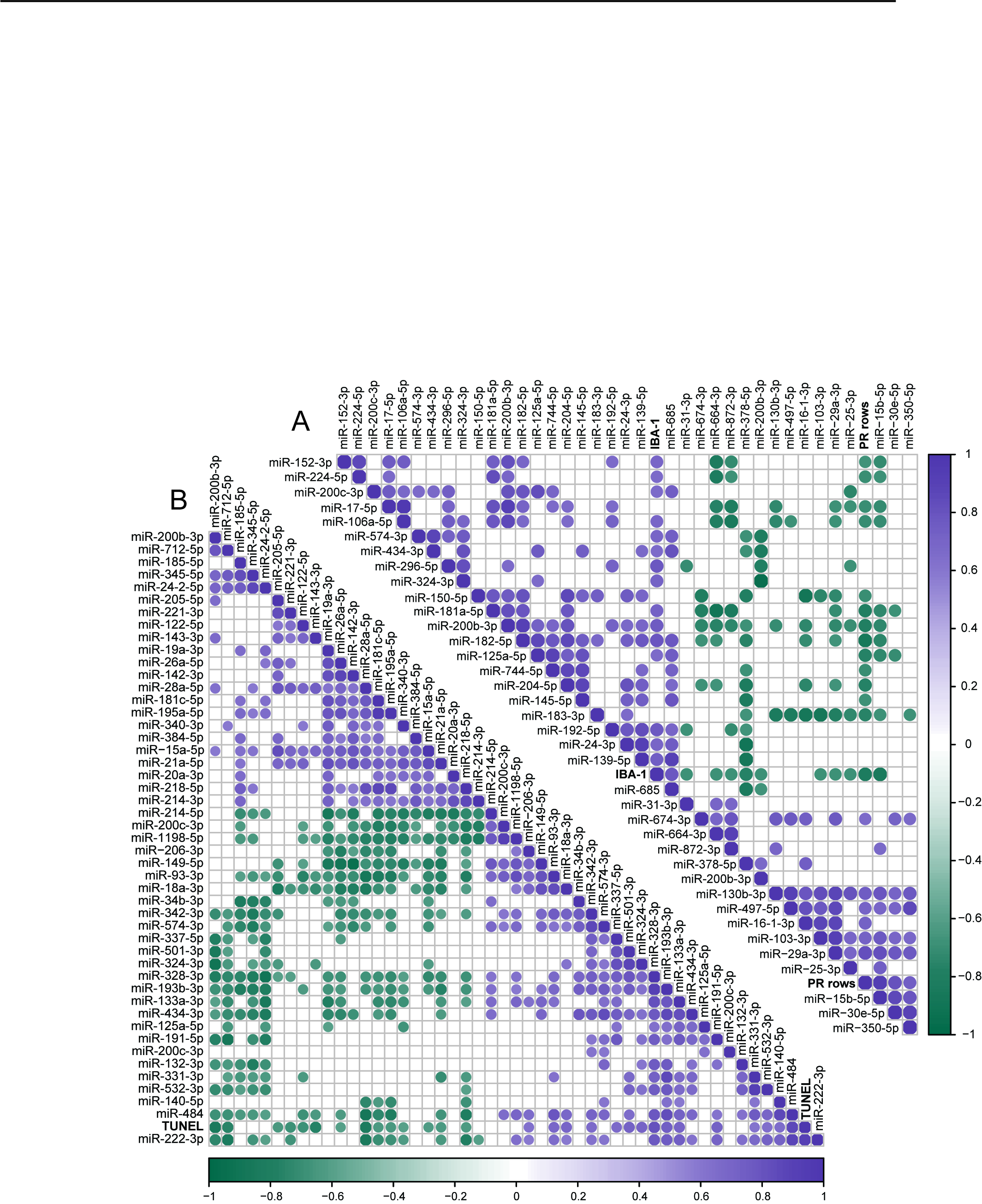

Supplement: Supplementary file 3 [file Image_2.TIF]

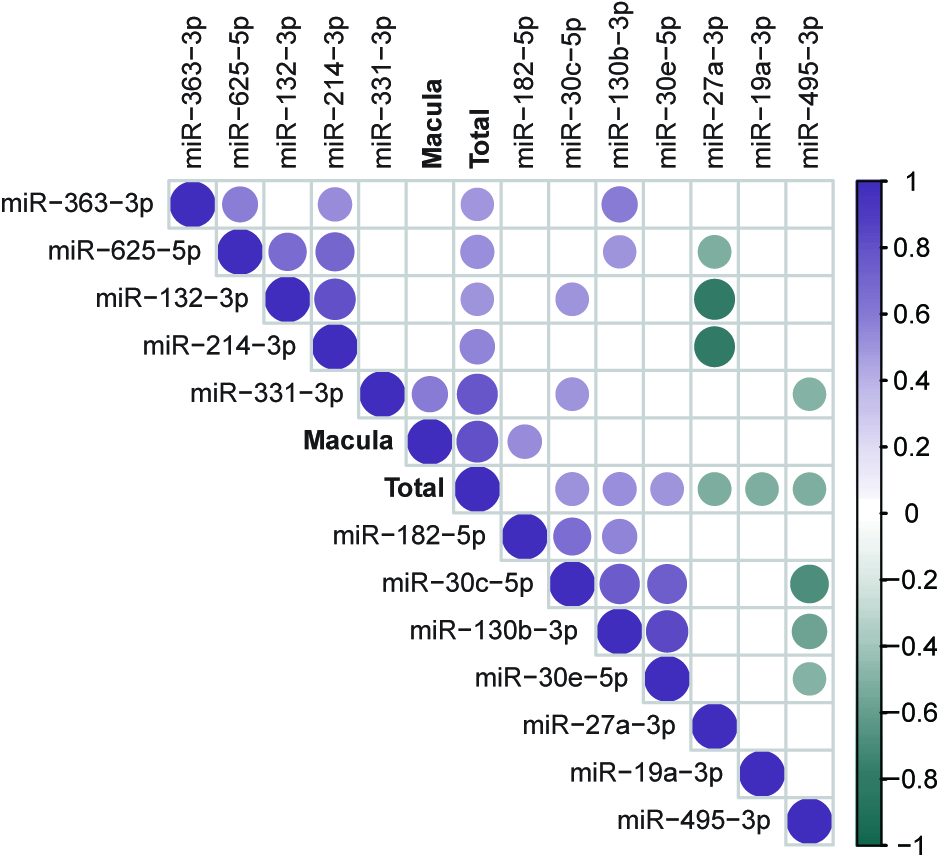

Supplement: Supplementary file 4 [file Image_3.TIF]
