# Supplementary material for: Serum miRNA modulations indicate changes in retinal morphology
Source: Front Mol Neurosci. 2023 Mar 3;16:1130249. doi: 10.3389/fnmol.2023.1130249 (PMC10020626; doi:10.3389/fnmol.2023.1130249)
Supplement: Supplementary file 5 [file Table_1.DOCX]

***Supplementary Table 1. Summary of details of patients recruited into this study***

|  |  | **Control** | **RPD** | **AMD** |
| --- | --- | --- | --- | --- |
| **Age** | 60-69 | 5 | 0 | 1 |
|  | 70-79 | 5 | 5 | 2 |
|  | >80 | 0 | 5 | 2 |
| **Sex** | Female | 4 | 8 | 4 |
|  | Male | 6 | 2 | 1 |
