# Supplementary material for: Serum miRNA modulations indicate changes in retinal morphology
Source: Front Mol Neurosci. 2023 Mar 3;16:1130249. doi: 10.3389/fnmol.2023.1130249 (PMC10020626; doi:10.3389/fnmol.2023.1130249)
Supplement: Supplementary file 6 [file Table_2.DOCX]

***Supplementary Table 2: Significant miRNA changes during photo-oxidative damage.***

| **A** | **miRNA** | **P-value** | **FC** |
| --- | --- | --- | --- |
|  | miR-218-5p | 0.01386802 | 0.76735405 |
|  | miR-128a-3p | 0.01525777 | 1.28343236 |
|  | miR-1-3p | 0.02532897 | 1.71928063 |
|  | miR-200b-3p | 0.02796555 | 0.99530877 |
|  | miR-410-3p | 0.03799423 | 5.8095392 |

| **B** | **miRNA** | **P-value** | **FC** |
| --- | --- | --- | --- |
|  | miR-574-3p | 0.00371864 | -1.2931991 |
|  | miR-200b-3p | 0.01161263 | 1.30513292 |
|  | miR-26a-5p | 0.01611418 | 1.05315522 |
|  | miR-434-3p | 0.02300556 | -0.7556376 |
|  | miR-126a-3p | 0.02628431 | 0.94133916 |
|  | miR-143-3p | 0.0411209 | 0.71990649 |
|  | miR-214-3p | 0.04201019 | -0.7826557 |

| **C** | **miRNA** | **P-value** | **FC** |
| --- | --- | --- | --- |
|  | miR-34b-3p | 1.85E-06 | 1.49767398 |
|  | miR-21a-5p | 6.22E-05 | -1.2383563 |
|  | miR-20a-5p | 8.16E-05 | -1.80888 |
|  | miR-342-3p | 9.79E-05 | 1.89789217 |
|  | miR-451a | 0.00020367 | -1.5277204 |
|  | miR-206-3p | 0.00034402 | 1.63185817 |
|  | miR-574-3p | 0.00047572 | 1.22779312 |
|  | miR-301a-3p | 0.00052198 | -1.4224704 |
|  | miR-106a-5p | 0.00058844 | -0.9926136 |
|  | miR-146b-5p | 0.00068352 | 1.26344444 |
|  | miR-25-3p | 0.00068596 | -1.2432566 |
|  | miR-26b-5p | 0.00068683 | -1.6002017 |
|  | miR-93-5p | 0.00086876 | 1.26989188 |
|  | miR-877-3p | 0.00124656 | 1.48303561 |
|  | miR-149-5p | 0.00127243 | 1.42889648 |
|  | let-7i-5p | 0.00172717 | -1.0800688 |
|  | miR-101b-3p | 0.00192838 | -0.8958235 |
|  | miR-93-3p | 0.00212235 | -1.1908595 |
|  | miR-193a-5p | 0.00216718 | 0.7503654 |
|  | miR-133a-3p | 0.00221044 | 1.58996777 |
|  | miR-195a-5p | 0.00251328 | -1.2941892 |
|  | miR-193b-3p | 0.0025539 | 0.98541816 |
|  | miR-664-3p | 0.00300393 | 1.10582379 |
|  | miR-296-5p | 0.00314676 | -1.2838335 |
|  | miR-125a-5p | 0.00324564 | 1.25120356 |
|  | miR-28a-5p | 0.00369453 | -1.012079 |
|  | miR-106b-5p | 0.00388291 | -1.2495518 |
|  | miR-18a-3p | 0.00404243 | 0.71637619 |
|  | miR-27a-3p | 0.00413219 | -0.7530132 |
|  | miR-142-3p | 0.00472417 | -1.560476 |
|  | miR-223-3p | 0.00511727 | 0.94761289 |
|  | miR-19b-3p | 0.0056616 | -1.0021259 |
|  | miR-27b-3p | 0.00753337 | -1.3992346 |
|  | miR-214-3p | 0.00776514 | 1.00756746 |
|  | miR-26a-5p | 0.00841192 | -1.4563446 |
|  | miR-130a-3p | 0.00869143 | -1.3994888 |
|  | miR-126a-3p | 0.01018514 | 0.89831962 |
|  | miR-181a-5p | 0.01029287 | -1.5811957 |
|  | miR-210-3p | 0.01059483 | -1.3051931 |
|  | miR-200c-3p | 0.01105661 | 0.60245975 |
|  | miR-146a-5p | 0.01185498 | 1.27939773 |
|  | miR-7a-3p | 0.01345061 | 0.58575353 |
|  | miR-146b-5p | 0.01576749 | 1.4239457 |
|  | miR-434-3p | 0.01638909 | 0.52299265 |
|  | miR-1839-5p | 0.01708945 | -1.5933555 |
|  | miR-186-5p | 0.01823383 | 0.97175235 |
|  | miR-674-3p | 0.01847323 | 0.57487056 |
|  | miR-139-5p | 0.02044182 | 0.60371321 |
|  | miR-1198-5p | 0.02170514 | 0.81906916 |
|  | miR-324-5p | 0.02389637 | -1.3547485 |
|  | miR-30d-5p | 0.02415058 | -1.5804724 |
|  | miR-126a-5p | 0.02848409 | -1.5171375 |
|  | miR-15a-5p | 0.02883369 | -1.4411582 |
|  | miR-365-3p | 0.03001428 | 0.89229976 |
|  | miR-700-3p | 0.03095182 | 0.62115226 |
|  | miR-191-5p | 0.03133242 | 0.54600734 |
|  | miR-22-5p | 0.0313852 | -1.5784536 |
|  | miR-148b-3p | 0.03500029 | -1.452507 |
|  | let-7d-5p | 0.035416 | -1.5192924 |
|  | miR-140-5p | 0.03739517 | -1.5117178 |
|  | miR-103-3p | 0.04439748 | -1.5360219 |
|  | miR-214-3p | 0.0468963 | 0.49174951 |
|  | miR-152-3p | 0.0496024 | -1.5908485 |
