# Supplementary material for: Serum miRNA modulations indicate changes in retinal morphology
Source: Front Mol Neurosci. 2023 Mar 3;16:1130249. doi: 10.3389/fnmol.2023.1130249 (PMC10020626; doi:10.3389/fnmol.2023.1130249)
Supplement: Supplementary file 9 [file Table_5.DOCX]

Supplementary Table 5. Significant miRNA changes between patients.

| 1. **RPD v Healthy** | **miRNA** | **p-value** | **FC** |
| --- | --- | --- | --- |
|  | hsa-miR-362-5p | 0.003414 | -1.37509 |
|  | hsa-miR-584-5p | 0.008089 | 1.928068 |
|  | hsa-miR-1249-3p | 0.021516 | 1.218508 |
|  | hsa-miR-483-3p | 0.026167 | 0.720361 |
|  | hsa-miR-17-5p | 0.030398 | -1.4775 |
|  | hsa-miR-21-5p | 0.033782 | -1.56028 |
|  | hsa-miR-155-5p | 0.040795 | -1.55781 |
|  | hsa-miR-29b-3p | 0.041732 | -1.37864 |
|  | hsa-miR-145-5p | 0.048015 | 0.785896 |

| 1. **GA v Healthy** | **miRNA** | **p-value** | **FC** |
| --- | --- | --- | --- |
|  | hsa-miR-21-5p | 9.27E-05 | -1.296 |
|  | hsa-miR-26a-5p | 0.001103 | -1.36795 |
|  | hsa-miR-584-5p | 0.001347 | 2.928877 |
|  | hsa-let-7g-5p | 0.002126 | -1.3674 |
|  | hsa-let-7d-5p | 0.002291 | -1.35309 |
|  | hsa-miR-1260a | 0.005026 | 1.763071 |
|  | hsa-let-7d-3p | 0.005651 | 1.416027 |
|  | hsa-miR-194-5p | 0.005849 | -1.4649 |
|  | hsa-miR-26b-5p | 0.008161 | -1.47661 |
|  | hsa-miR-331-3p | 0.013515 | 1.950798 |
|  | hsa-miR-19b-3p | 0.013814 | -1.47028 |
|  | hsa-miR-103a-2-5p | 0.013875 | 0.66785 |
|  | hsa-miR-19a-3p | 0.015437 | -1.37479 |
|  | hsa-miR-30c-5p | 0.016194 | 0.587071 |
|  | hsa-let-7i-5p | 0.016387 | -1.43306 |
|  | hsa-miR-107 | 0.018861 | -1.36851 |
|  | hsa-miR-652-3p | 0.020104 | -1.4072 |
|  | hsa-miR-340-5p | 0.020105 | 1.18706 |
|  | hsa-miR-374a-5p | 0.02269 | -1.43336 |
|  | hsa-miR-96-5p | 0.023104 | 0.962411 |
|  | hsa-miR-18a-5p | 0.02404 | -1.46483 |
|  | hsa-miR-29c-5p | 0.024544 | -1.48692 |
|  | hsa-miR-625-5p | 0.025792 | 1.769693 |
|  | hsa-miR-433-3p | 0.026275 | 5.171496 |
|  | hsa-miR-15b-3p | 0.027699 | 1.63629 |
|  | hsa-miR-27a-3p | 0.028159 | -1.4219 |
|  | hsa-miR-126-5p | 0.029258 | -1.46652 |
|  | hsa-miR-17-5p | 0.031708 | -1.50737 |
|  | hsa-miR-324-3p | 0.031978 | 1.227436 |
|  | hsa-miR-574-3p | 0.031983 | 2.892388 |
|  | hsa-miR-505-5p | 0.03419 | 4.989772 |
|  | hsa-miR-155-5p | 0.034822 | -1.55036 |
|  | hsa-miR-30b-5p | 0.035488 | 0.392487 |
|  | hsa-miR-137 | 0.03565 | 1.878371 |
|  | hsa-miR-374b-5p | 0.037367 | -1.45222 |
|  | hsa-let-7f-2-3p | 0.040702 | 0.530869 |
|  | hsa-miR-483-3p | 0.041166 | 0.686771 |
|  | hsa-miR-375 | 0.043202 | 1.2854 |
|  | hsa-miR-143-3p | 0.044478 | 0.539628 |
|  | hsa-miR-653-5p | 0.044519 | 0.793928 |
|  | hsa-miR-29b-3p | 0.044945 | -1.38007 |
|  | hsa-miR-1-3p | 0.046293 | 0.918185 |
|  | hsa-miR-93-3p | 0.046722 | 0.377148 |

| 1. **GA v RPD** | **miRNA** | **p-value** | **FC** |
| --- | --- | --- | --- |
|  | hsa-miR-30c-5p | 0.005053 | 0.701422 |
|  | hsa-miR-625-5p | 0.006268 | 2.424189 |
|  | hsa-miR-340-5p | 0.007555 | 1.396156 |
|  | hsa-miR-21-5p | 0.007731 | -1.4978 |
|  | hsa-miR-126-5p | 0.009035 | -1.41388 |
|  | hsa-miR-331-3p | 0.010919 | 1.870222 |
|  | hsa-miR-7-1-3p | 0.011703 | 2.406129 |
|  | hsa-miR-26b-5p | 0.01256 | -1.5097 |
|  | hsa-let-7g-5p | 0.013159 | -1.48117 |
|  | hsa-miR-574-3p | 0.01345 | 3.552104 |
|  | hsa-miR-7-5p | 0.013614 | 0.709748 |
|  | hsa-miR-532-3p | 0.018438 | 0.439299 |
|  | hsa-miR-505-5p | 0.019368 | 5.513784 |
|  | hsa-miR-30b-5p | 0.020343 | 0.418493 |
|  | hsa-miR-26a-5p | 0.023159 | -1.53625 |
|  | hsa-miR-30e-3p | 0.024009 | 1.977322 |
|  | hsa-miR-15b-5p | 0.027858 | -1.38646 |
|  | hsa-miR-548k | 0.028986 | 2.040552 |
|  | hsa-miR-576-5p | 0.02924 | 0.399759 |
|  | hsa-miR-579-3p | 0.030486 | 11.67581 |
|  | hsa-let-7d-5p | 0.032154 | -1.52598 |
|  | hsa-miR-29c-5p | 0.039671 | -1.53807 |
|  | hsa-miR-328-3p | 0.041663 | 0x.343124 |
|  | hsa-miR-652-3p | 0.043656 | -1.48309 |
|  | hsa-miR-495-3p | 0.047386 | -1.61259 |
|  | hsa-miR-185-5p | 0.049397 | -1.49578 |
